# Population pharmacokinetic analysis of rivaroxaban in healthy volunteers and patients with radiofrequency ablation of non-valvular atrial fibrillation in China

**DOI:** 10.3389/fphar.2025.1562259

**Published:** 2025-05-29

**Authors:** Fangfang Gu, Kaixian Tang, Cong Zhang, Mingsheng Hu, Junlong Sun, Xiang Yu, Mengli Tian, Chen Zhang, Yingrong Chen

**Affiliations:** ^1^ Department of Cardiology, Huzhou Central Hospital, Fifth School of Clinical Medicine of Zhejiang Chinese Medical University, Huzhou, China; ^2^ Clinical Trial Center, Huzhou Central Hospital, Fifth School of Clinical Medicine of Zhejiang Chinese Medical University, Huzhou, China; ^3^ Huanzhu Longquan Street Community Health Service Center, Huzhou, China

**Keywords:** rivaroxaban, population pharmacokinetics, modeling and simulation, non-valvular atrial fibrillation, Chinese population

## Abstract

**Aims:**

This study aimed to develop a population pharmacokinetic (PopPK) model of rivaroxaban in healthy volunteers and patients with radiofrequency ablation of non-valvular atrial fibrillation (NVAF) in China and investigate the effect of potential covariates on pharmacokinetic (PK) parameters.

**Methods:**

Plasma concentrations of rivaroxaban with demographic data, biochemical indicators, and genetic data were derived from a bioequivalence study in 36 healthy volunteers and a real-world study containing 105 patients with NVAF. A PopPK model of rivaroxaban was performed with NONMEM software using a nonlinear mixed-effect modeling approach, and covariate impact on rivaroxaban pharmacokinetics was investigated.

**Results:**

A two-compartment model characterized by first-order absorption and first-order linear elimination successfully described the pharmacokinetic properties of rivaroxaban. In the final PopPK model, the clearance rate for patients was 8.35 L/h, and the central and peripheral volumes of distribution were 19.7 L and 71.8 L, respectively. The creatinine clearance, ABCB1 rs1045642, and morbid state were identified as significant covariates affecting the clearance of rivaroxaban. The AUC_0-inf_ increased by 58% for patients with moderate renal impairment compared to subjects with normal renal function. The AUC_0-inf_ for patients with the wild genotype of ABCB1 rs1045642 was 25% higher than that for other genotypes. The validation results demonstrated the good predictability of the model, which was accurate and reliable.

**Conclusion:**

The PopPK model of rivaroxaban in healthy volunteers and patients with NVAF developed in this study was expected to help provide relevant PK parameters and covariate information for further studies of rivaroxaban. The study indicated that a daily dose of 15 mg may be appropriate as the primary dosage of rivaroxaban for Chinese patients with NVAF. A lower dose is recommended for patients with moderate renal impairment to avoid overexposure.

## 1 Introduction

Non-valvular atrial fibrillation (NVAF) is one of the most prevalent cardiac arrhythmias worldwide, significantly contributing to the incidence of stroke, thromboembolism, heart failure, and myocardial infarction (Benjamin et al., 2019; January et al., 2014). Patients with NVAF are at a significantly increased risk of ischemic stroke, estimated to be four-to-five-times higher than in patients with sinus rhythm (Wolf et al., 1991). In recent years, catheter ablation and novel oral anticoagulants (NOACs) have significantly transformed the treatment of atrial fibrillation (Kirchhof et al., 2016; Patel et al., 2019). Radiofrequency ablation (RFA) has emerged as the most effective treatment for NVAF, offering significant advantages over antiarrhythmic medications in terms of maintaining sinus rhythm, reducing the burden of NVAF, alleviating symptoms, and enhancing exercise tolerance (Calkins et al., 2009; Hakalahti et al., 2015).

The primary causes of mortality in patients with NVAF include stroke, progressive heart failure, and cardiac arrest. Consequently, the prevention of thromboembolic events associated with NVAF through appropriate anticoagulation therapy is a critical component of its management. Oral anticoagulant therapy is recommended for all patients with NVAF following surgery. It should be initiated as soon as possible and maintained for a minimum of 2–3 months (Trujillo and Dobesh, 2014). Rivaroxaban, a direct factor Xa inhibitor, is used for the prevention of thromboembolic events in patients with NVAF. It is rapidly absorbed and reaches peak plasma concentration within 2–4 h after oral administration (Mueck et al., 2014). Rivaroxaban exhibits high plasma protein binding (92%–95%) and a distribution volume at a steady state of approximately 50 L (0.62 L/kg) (Mueck et al., 2014). Approximately 35% of the rivaroxaban dose is excreted renally, primarily involving gene-encoded transporters P-glycoprotein (ABCB1) and breast cancer resistance protein (ABCG2) in the active renal secretion process (Kubitza et al., 2010). The remaining 65% of rivaroxaban is metabolized by the liver, with several cytochrome P450 (CYP) enzymes, including CYP3A4/5 and CYP2J2, being responsible for its metabolism (Mueck et al., 2013; Kvasnicka et al., 2017). Age and renal function have been shown to influence rivaroxaban clearance; specifically, clearance decreases with advancing age, and the volume of distribution is affected by both age and body size. For elderly patients, the elimination half-life is 11–13 h, compared to 5–9 h for younger patients (Mueck et al., 2011). Previous research has indicated that various factors, including renal impairment, the use of CYP3A4 or P-glycoprotein inhibitors, age, gender, and body weight, may impact the pharmacokinetic parameters of rivaroxaban (Kanuri and Kreutz, 2019). Therefore, it is essential to investigate the factors influencing the variability in rivaroxaban levels among patients with NVAF to ensure its appropriate use.

Population pharmacokinetics (PopPK) is widely used to characterize the pharmacokinetics of the drug under investigation and to evaluate the potential factors contributing to pharmacokinetic variability within the population. PopPK models of rivaroxaban have been developed across various conditions, including NVAF, acute coronary syndrome (ACS), venous thrombosis, and hip/knee replacement surgery (Zhang et al., 2022; Zdovc et al., 2019; Mueck et al., 2008; Mueck et al., 2011; Zhao et al., 2022), to identify significant covariates that support rational clinical application or individualized administration. Until now, there have been no reports on PopPK studies of rivaroxaban for both healthy volunteers and patients with radiofrequency ablation of NVAF that quantitatively evaluated the influence of genetics.

The present study used nonlinear mixed-effects modeling to develop and optimize the first PopPK model of rivaroxaban in both healthy volunteers and patients with radiofrequency ablation of NVAF in China. We aimed to quantitatively analyze the influence of demographic, genetic, and clinical data and concomitant medication on drug PK parameters and provide support for the individualized medication of rivaroxaban in clinical practice.

## 2 Methods

### 2.1 Study design and analytical method

#### 2.1.1 Study 1

This study was a single-center, single-dose randomized, two-formulation, four-cycle repeat crossover trial (No. CTR20202135). Thirty-six healthy Chinese volunteers were randomized into two groups and administered a single oral dose of 20 mg of the test or reference formulation of rivaroxaban tablets per cycle, following a fasting period of at least 10 h. The washout period between each cycle was 7 days. The concentrations of rivaroxaban were included during the cycles of the reference drug (Xarelto, Bayer). Eighteen blood samples (4 mL) were collected pre-dose and at 0.25 h, 0.5 h, 0.75 h, 1.0 h, 1.5 h, 2.0 h, 2.5 h, 3.0 h, 3.5 h, 4.0 h, 5.0 h, 6.0 h, 8.0 h, 14.0 h, 24.0 h, 36.0 h, and 48.0 h post dose in each period. Plasma samples were directly separated by centrifugation at 1,700 × g for 10 min at 4°C, transferred into polypropylene plastic tubes, and stored at −60°C to −90°C.

A validated LC-MS/MS method was used to determine the concentration of rivaroxaban in human plasma (Tao et al., 2020). The analytes were chromatographed on a Shim-pack GIST C8 column (Shimadzu, Japan), and detection was performed using the LCMS-8050 tandem mass spectrometer and LC-30AD high-performance liquid chromatography system coupled with an electrospray ionization (ESI) source (Shimadzu, Japan). Rivaroxaban concentration remained linear within the range of 1–600 ng/mL, with a lower limit of quantification of 1 ng/mL. Limits of precision and accuracy for calibrators and quality controls (QCs) were ±20% at the lower limit of quantification (LLOQ) and ±15% at other concentration levels. The matrix effect remained consistent among QCs. No endogenous interferents were detected at the retention times of rivaroxaban and the internal standard of rivaroxaban-d4. Data points below the LLOQ during the absorption phase were assigned a value of zero and excluded from statistics during the elimination phase.

Ten single-nucleotide polymorphisms (SNPs) of cytochrome P450 and drug transporter genes were genotyped by polymerase chain reaction. Sanger sequencing was performed using a 3730XL DNA analyzer, and the primer sequences are shown in the electronic supplementary material (ESM) (Supplementary Table S1).

#### 2.1.2 Study 2

This study was a real-world, single-center study involving Chinese patients undergoing radiofrequency ablation of NVAF at Huzhou Central Hospital (No. ChiCTR2500095918). Patients who received radiofrequency ablation of non-valvular atrial fibrillation and were hospitalized between April 2023 and November 2023 taking doses of rivaroxaban (Xarelto, Bayer) at 10–20 mg once daily were enrolled. Following a minimum of 2 days of rivaroxaban administration, blood samples were collected 30 min before medication and 2–4 h after medication. Throughout the study, demographic data, including age, weight, height, sex, duration of current therapy, concomitant drug therapy, comorbidities, and rivaroxaban dosing regimens, were recorded. Additionally, biochemical parameters, including routine blood tests, liver renal function, and blood coagulation indexes, were also obtained.

The simultaneous determination of rivaroxaban was conducted by LC-MS/MS. The linearity ranges were 1–600 ng/mL for rivaroxaban. Analytes were extracted by protein precipitation using acetonitrile. Deuterated internal standards of rivaroxaban-d4 were used. The linearity, sensitivity, matrix effect, extraction efficiency, accuracy, precision, and stability were validated and acceptable. Data beyond quantitative ranges were labeled and excluded in this study. Ten single-nucleotide polymorphisms (SNPs) of cytochrome P450 and drug transporter genes were also genotyped.

### 2.2 Population PK model development

The PopPK model was constructed using a nonlinear mixed-effects modeling tool NONMEM (version 7.5.0, ICON Development Solutions, Ellicott City, Maryland, United States). Perl Speaks NONMEM (PsN) Ver 4.2.3 (Uppsala University, Sweden) (Lindbom et al., 2004) and R ver. 4.1.1 were used in the study (R Core Team, 2022). All models were fitted using the first-order conditional estimation (FOCE) method with interaction (Lindstrom and Bates, 1990).

During the modeling process, one-compartment, two-compartment, and three-compartment models will be considered structural models, and nonlinear absorption or elimination models will also be considered when necessary. Once the structural model is established, individual variability of parameters and correlations between parameters [usually Clearance rate (CL) and Central volume of distribution (Vc)] will be further investigated.

The interindividual variability (IIV) of each PK parameter was assessed using the following equation:

$$\theta_{i}=e^{\left(\theta_{T}+\eta_{i}\right),}$$

where *θi* is the PK parameter for the *i*th subject, *θ*_*T*_ is the typical population value of the PK parameter, and *ηi* represents the random interindividual variability, which follows a normal distribution with a mean of 0 and variance of *ω* ^*2*^. Residual variability (RV) was described as follows:

$$C\left(t\right)\mathrm{ij}=Ĉ\left(t\right)\mathrm{ij}*\left(1+\varepsilon_{1\mathrm{ij}}\;\right)+\varepsilon_{2\mathrm{ij},}$$

where *C(t)*_*ij*_ and *Ĉ(t)*_*ij*_ represent the *j*th observed and model-predicted concentrations for the *i*th subject, respectively, and *ε*_*1ij*_ and *ε*_*2ij*_ are the proportional and additive residual errors for the *j*th observed concentration of the *i*th subject, which are independent and follow normal distributions with means of 0 and variances of *σ*_*1*_^*2*^ and *σ*_*2*_^*2*^, respectively.

Covariate selection was conducted using common forward and backward methods, with *p*-values of 3.84 and 6.63 for the forward and backward methods, respectively (corresponding to *p* = 0.05 and 0.01 for 1 degree of freedom). The model for the impact of continuous and categorical covariates on PK parameters is as follows:

$$\theta_{i}=\theta_{T}\cdot \exp\left(k_{Cov}\cdot \ln\left(\frac{Cov_{i}}{Cov_{pop}}\right)+\eta_{i}\right),$$

$$\theta_{i}=e^{{\theta_{T}+k}_{cov}*X_{i}}$$

where *θi* is the PK parameter for the *i*th subject; *θ*_*T*_ is the typical population value of the log-transformed PK parameter; *Cov*_*i*_ is the continuous covariate value for the *i*th subject; *Cov*_*pop*_ is the median of the continuous variable in the population; *X*_*i*_ is the indicator for the categorical variable for the *i*th subject, where a value of 0 represents the most common category of the covariate, and other integer values represent other categories; *k*_*cov*_ is the coefficient describing the magnitude of the covariate effect; and *ηi* represents the random interindividual variability following a normal distribution with a mean of 0 and variance of *ω* ^*2*^.

The covariates in the demographic data encompass gender (SEX), age (AGE), height (HT), and weight (WT). Additionally, hepatic and renal function covariates, along with blood biochemistry-related variables, include white blood cell count (WBC), red blood cell count (RBC), platelet count (PLT), glucose (GLU), creatine (Cr), alanine aminotransferase (ALT), aspartate aminotransferase (AST), calcium (CA), potassium (K), creatine kinase (CK), and creatinine clearance rate (CRCL). Genetic polymorphism covariates incorporated in the selection process include CYP3A4 rs2242480, rs2246709, rs3735451, CYP3A5 rs776746, ABCB1 rs1045642, rs1128503, rs2032582, rs4148738, rs4728709, and ABCG2 rs3114018.

### 2.3 Population PK model evaluation

The model’s prediction accuracy in correlating concentrations with observed values was assessed through a goodness of plot, which included comparisons of population predicted concentrations (PRED) to observed concentrations (DV), individual predicted concentrations (IPRED) to DV, conditional weighted residuals (CWRES) to PRED, and a correlation plot of CWRES against time after the previous dose (TAD). The predictive capability of the final model in characterizing the pharmacokinetic profile of rivaroxaban was evaluated using the visual predictive check (pcVPC) method. The pcVPC involved simulating 1,000 trials based on the final model parameters, random effects, residuals, individual covariates, and actual dosing regimens. The predicted outcomes were compared with observed values through graphical representation, allowing for an assessment of the alignment between the median and the distribution range (2.5th–97.5th percentiles) of both observed and model-predicted pharmacokinetic profiles. The stability of the final model was examined using the bootstrap method, wherein 1,000 datasets were generated with the same number of subjects as the original dataset, and each dataset was sequentially refitted with the final model to derive 1,000 sets of model parameters. Statistical comparisons were conducted between these 1,000 model parameters and the parameters obtained from the final model.

### 2.4 Influence of covariates on rivaroxaban pharmacokinetics

When the final model is established, further analysis will focus on significant covariates. The influence of various covariate subgroups on AUC_0-inf_ will be assessed. AUC_0-inf_ will be calculated using the formula dose/CL, with all subjects receiving a consistent dose of 15 mg. Initially, a reference geometric mean exposure level will be determined for a group of subjects, and the geometric mean exposure levels of different subgroups will be compared to that of the reference group. The extent of the impact will be visually represented through forest plots.

## 3 Results

### 3.1 Baseline of demographics and clinical characteristics

A total of 1,296 concentrations from 36 healthy volunteers and 210 concentrations from 105 patients with radiofrequency ablation of NVAF (105 trough concentrations and 105 peak concentrations) were included for modeling. Baseline demographics and clinical characteristics are summarized in Table 1. Ten gene loci were detected in 36 healthy volunteers and 105 patients, with a total of 1,410 gene data. The distributions of the genotypes agreed with the Hardy–Weinberg equilibrium, with the exception of ABCB1 rs2032582, rs4148738, and rs4728709 in healthy volunteers and ABCB1 rs2032582 in patients. The detailed allele frequencies are shown in Table 2.

**TABLE 1:** Baseline of demographics and clinical characteristics.

	Study Ⅰ (n=36)	Study Ⅱ (n=105)
Dose(mg)		
10	0 (0%)	2 (1.9%)
15	0 (0%)	101 (96.2%)
20	36 (100%)	2 (1.9%)
Sex(%)		
Male	32 (88.89%)	60 (57.14%)
Female	4 (11.11%)	45 (42.86%)
AGE, years (range)	31.6±8.9 (18, 48)	64.1±8.4 (34, 82)
WT, kg (range)	64.2±5.8 (53.2, 76.8)	67.4±11.4 (42.0, 107.0)
HT (cm)	167±7 (153, 182)	165±8 (145,183)
WBC, 10^9^/L (range)	6.1±1.3 (3.3, 9.5)	6.0±1.6 (2.8, 10.5)
RBC, 10^12^/L (range)	5.0±0.4 (4.1, 5.8)	4.4±0.5 (3.3, 5.7)
PLT, 10^9^/L (range)	233.8±44.3 (151.0, 333.0)	186.0±58.8 (90.0, 514.0)
GLU, mmol/L (range)	4.8±0.3 (4.0, 5.3)	5.0±0.9 (3.8, 9.4)
Cr,μmol/L (range)	69.1±6.67 (56.3, 95.0)	75.3±18.5 (52.0, 145.1)
CRCL, ml/min (range)^a^	125±17.8 (91.3, 161)	86.7±24.3 (33.6, 158)
ALT, U/L (range)	20.1±7.8 (11.4, 38.5)	27.8±21.1 (9.4, 134.3)
AST, U/L (range)	19.6±4.5 (15.2, 35.7)	24.5±10.2 (14.6, 77.2)
Ca, mmol/L (range)	2.3±0.1 (2.1, 2.5)	2.2±0.1 (2.0, 2.5)
K, mmol/L (range)	4.2±0.3 (3.7, 4.7)	3.8±0.4 (3.0, 5.1)
CK, U/L (range)	95.8±33.8 (37.3, 195.1)	84.1±47.6 (24.5, 316.6)
Concomitant medication		
CYP3A4 inhibitor (%)	-	6 (5.7%)
P-gp inhibitor (%)	-	56 (53.3%)

WT, Weight; HT, height; WBC, white blood cell; RBC, red blood cell; PLT, platelet; GLU, glucose; Cr, creatine; CrCL,creatinine clearance; ALT, alanine aminotransferase; AST, aspartate aminotransferase;

^a^ Creatinine clearance (CRCL) was calculated by Cockcroft-Gault formula: CRCL (ml/min)=[140 – Age (year)] × BW (kg) × 0.85 (if female)/[72 × Cr (mg/dl)].

**TABLE 2:** Genotype frequency and allele distribution.

Gene	SNPs	Genotype	Study Ⅰ (n=36)	p-Value (Hardy-Weinberg equilibrium)	Study Ⅱ (n=105)	p-Value (Hardy-Weinberg equilibrium)
CYP3A4	rs2242480	TT	4 (11.1%)	0.449	7 (6.7%)	0.668
		CT	13 (36.1%)		37 (35.2%)	
		CC	19 (52.8%)		61 (58.1%)	
	rs2246709	AG	14 (38.9%)	0.275	52 (49.5%)	0.679
		GG	7 (19.4%)		15 (14.3%)	
		AA	15 (41.7%)		38 (36.2%)	
	rs3735451	CC	4 (11.1%)	0.450	8 (7.6%)	0.655
		CT	19 (52.8%)		45 (42.9%)	
		TT	13 (36.1%)		52 (49.5%)	
CYP3A5	rs776746	TT	3 (8.3%)	0.777	8 (7.6%)	0.896
		CT	16 (44.4%)		41 (39.0%)	
		CC	17 (47.2%)		56 (53.3%)	
ABCB1	rs1045642	GG	18 (50.0%)	5.05×10^-2^	42 (41.0%)	0.142
		AG/CG	11 (30.6%)		43 (40.0%)	
		AA	7 (19.4%)		20 (19.0%)	
	rs1128503	AG	20 (55.6%)	0.391	40 (38.1%)	0.0592
		AA	11 (30.6%)		46 (43.8%)	
		GG	5 (13.9%)		19 (18.1%)	
	rs2032582	CC/TT	19 (52.8%)	6.35×10^-7^	27 (25.7%)	0.0186
		AA	14 (38.9%)		38 (36.2%)	
		AC/AT/CT	3 (8.3%)		40 (38.1%)	
	rs4148738	TT	18 (50.0%)	1.69×10^-2^	37 (35.2%)	0.0813
		CC	8 (22.2%)		25 (23.8%)	
		CT	10 (27.8%)		43 (41.0%)	
	rs4728709	GG	23 (63.9%)	4.86×10^-3^	77 (73.3%)	0.909
		AG	13 (36.1%)		26 (24.8%)	
		AA	0 (0%)		2 (1.9%)	
ABCG2	rs3114018	AC	17 (47.2%)	0.965	50 (47.6%)	0.997
		CC	14 (38.9%)		39 (37.1%)	
		AA	5 (13.9%)		16 (15.2%)	

### 3.2 Population PK model development

The final PopPK structural model for rivaroxaban selected a two-compartment model characterized by first-order absorption and first-order linear elimination. The PopPK model parameters encompass the central clearance rate (CL/F), central volume of distribution (Vc/F), absorption rate constant (K_a_), inter-compartmental clearance rate (Q/F), peripheral volume of distribution (Vp/F), absorption lag time (ALAG1), and bioavailability (F). During the base modeling process, variations in bioavailability across different dose groups were examined, as well as potential differences between patients and healthy individuals.

To mitigate the impact of these differences on subsequent covariate screening results, these factors were thoroughly investigated at the base model stage. The findings revealed significant disparities in bioavailability among the various dose groups. The bioavailability of the most commonly administered 15 mg dose group was defined as 1, with the estimated relative bioavailability of the 10 mg dose group calculated at 1.363. Given the limited sample size in the 10 mg group, this parameter was fixed and not estimated in the subsequent models. The relative bioavailability of the 20 mg dose group was determined to be 0.537. The morbid state was identified as a significant factor influencing the clearance, with patients exhibiting a slightly higher clearance.

During the forward selection process for covariate screening, CRCL demonstrated the most significant impact on CL and was, therefore, included in the model first. Subsequently, the influence of ABCB1 rs1045642, treated as a four-category variable, on CL, along with the effect of weight on Vp, proved significant, resulting in the inclusion of these variables in the full model. In the following backward elimination process, the influence of weight did not satisfy the criteria for retention and was consequently removed. Notably, significant differences were observed primarily between the AA genotype and the other three groups within the four-category variable of ABCB1 rs1045642. As a result, ABCB1 rs1045642 was reclassified into a two-category variable (AA vs Non-AA). CRCL remained significant and was retained throughout the backward elimination process. None of the other covariates were significant. In the final model parameter estimates, the relative bioavailabilities for the 10 mg, 15 mg, and 20 mg doses were 1.363, 1, and 0.537, respectively; the clearance rate for patients was 8.35 L/h, while for healthy individuals, it was 6.48 L/h; the central and peripheral volumes of distribution were 19.7 L and 71.8 L, respectively; the inter-compartmental clearance rate was 7.64 L/h; and the absorption rate and absorption lag time were 0.46 1/h and 0.168 h, respectively. CRCL and the AA genotype of ABCB1 rs1045642 were significant covariates on clearance. The final PK parameters and covariate relationships equation derived from this analysis are provided below:

$${CL}_{i}=TVCL*\exp\left(1.53*\ln\left(\frac{{CRCL}_{i}}{97.7}\right)-AA*0.204+\eta_{CL}\right),$$

$$V_{2i}=19.7*\exp\left(\eta_{V2}\right),$$

$$K_{ai}=0.46*\exp\left(\eta_{ka}\right),$$

$$Q_{i}=7.64*\exp\left(\eta_{Q}\right),$$

$$V_{3i}=71.8*\exp\left(\eta_{V3}\right),$$

$${ALAG}_{1i}=0.168*\exp\left(\eta_{ALAG1}\right).$$

$F_{1i}=1$ for 15 mg, $F_{1i}=1.363$ for 10 mg, $F_{1i}=0.537$ for 20 mg; TVCL = 6.48 for healthy volunteer, TVCL = 8.35 for patient; AA = 1 for AA genotype of rs1045642, AA = 0 for other genotypes of rs1045642.

### 3.3 Final population PK model evaluation

**FIGURE 1:**
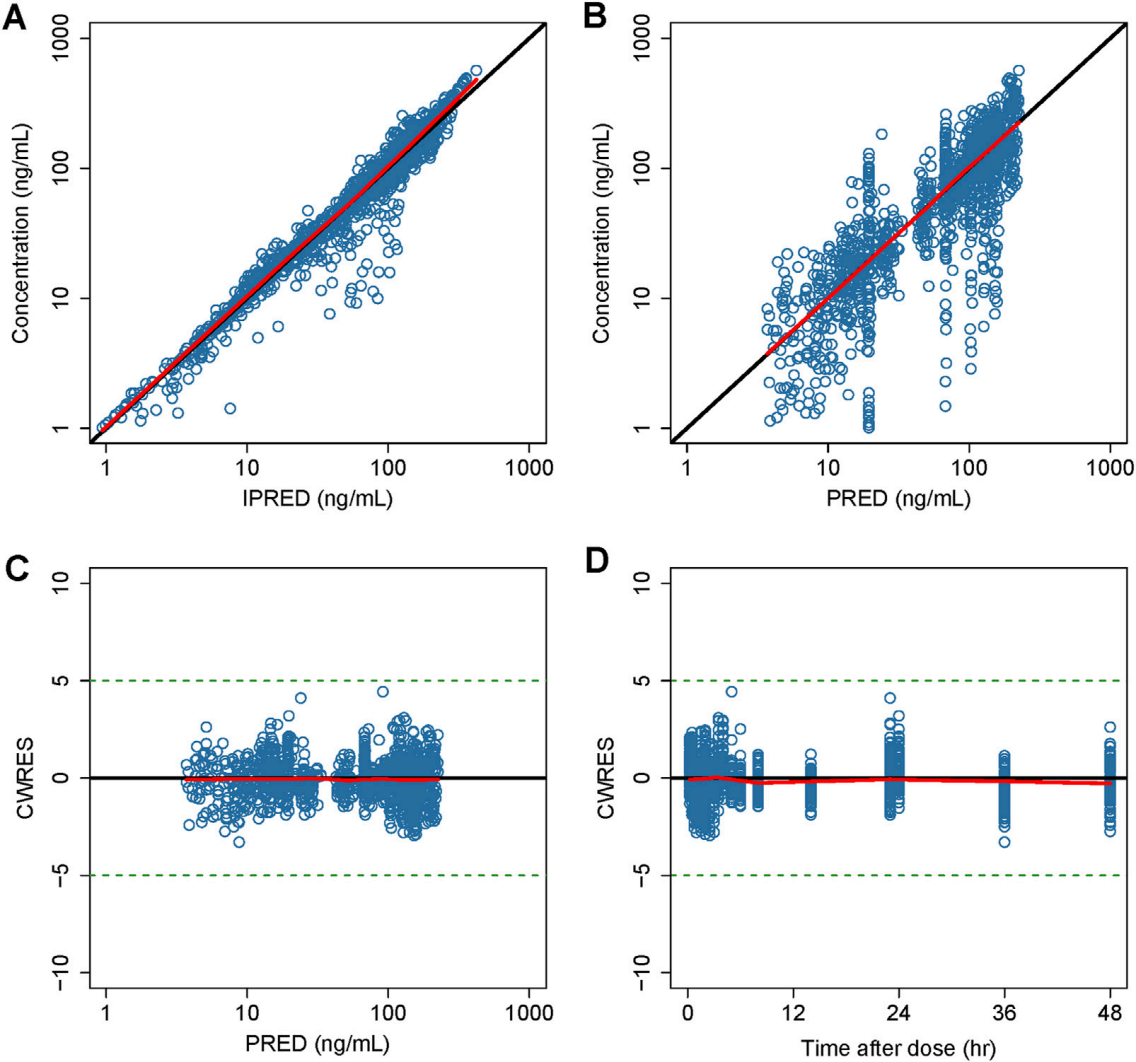
Goodness-of-fit plot of the final model. **(A)** Observed concentrations versus IPERD. **(B)** Observed concentrations versus PRED. **(C)** CWRES versus PRED. **(D)** CWRES versus time. Black lines represent the identity lines in **(A,B)**, while in **(C)** and **(D)**, they represent the position where CWRES equals 0. Red lines represent the nonparametric regression lines.

**FIGURE 2:**
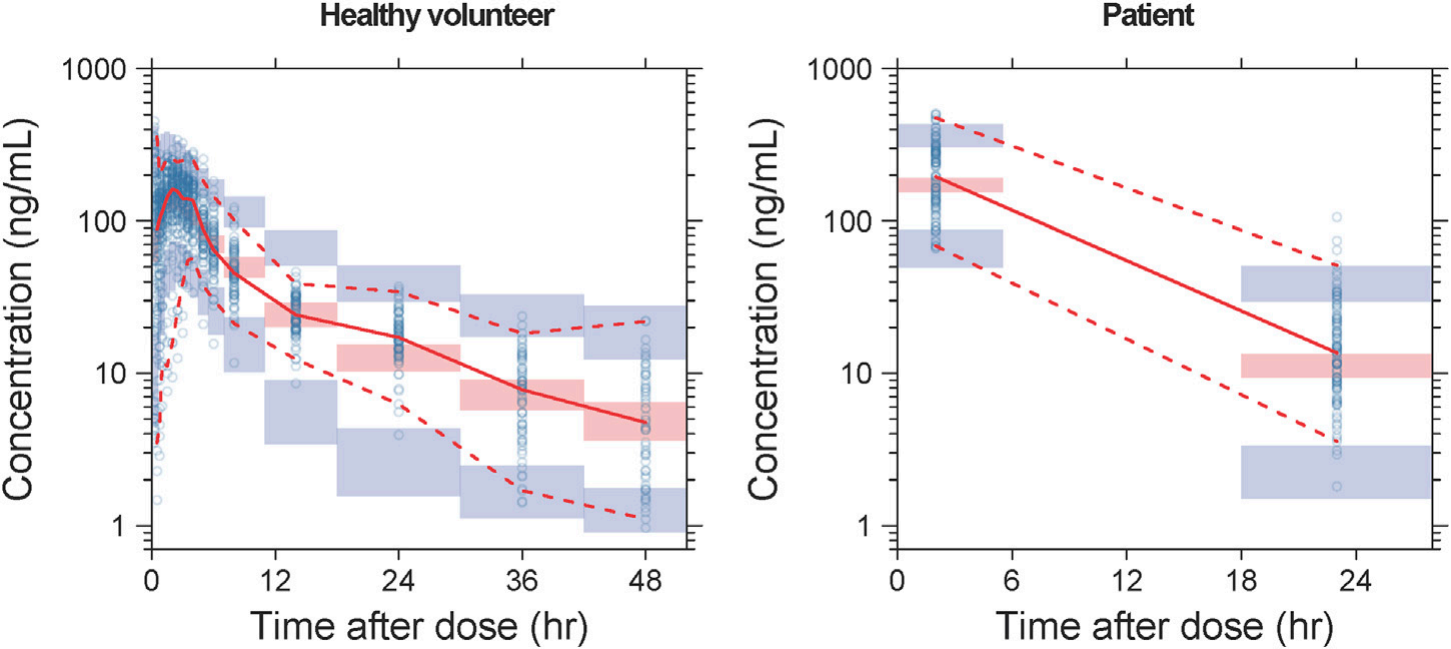
pcVPC results of the final model. The solid line and dashed lines, respectively, represent the median and 95% CI of the observations. Open circles represent the observed concentrations. The shaded red area represents the 95% CI of medians, and the shaded blue areas stand for the 95% CI of the 2.5th and 97.5th percentiles of the simulation results.

Detailed parameter estimates for the final model are presented in Table 3. All the relative standard error (RSE)% values of the structure model parameter were within 20%, indicating these parameters were well estimated. The model diagnostic plots are illustrated in Figure 1 which indicates a very good model fit with no evident trend bias. The pcVPC plot (Figure 2) displayed concentration observations for all subjects, along with the 95% confidence interval of the model-predicted median and the 95% confidence intervals for the model-predicted 2.5th and 97.5th percentiles. These findings suggested that the final population pharmacokinetic model effectively captures the concentration trends and variability of rivaroxaban in subjects from clinical studies. Bootstrap validation results for the final model are also detailed in Table 3. The median values derived from 1,000 bootstrap iterations closely align with the parameter estimates, indicating strong model stability. The shrinkage values for CL, V_2_, K_a_, Q, V_3_, and ALAG were 14%, 27.2%, 56.6%, 24.8%, 36.3%, and 45.9%, respectively. Among these parameters, K_a_, ALAG, and V_3_ showed higher shrinkage, likely due to the predominance of patients in the dataset and the sparse sampling points. Notably, the shrinkage for the two critical parameters, CL and V_2_, remained below 30%.

**TABLE 3:** Population PK parameters of rivaroxaban and bootstrap results.

Parameter	Parameter Description	Estimate (% RSE)	Bootstrap Estimates Median (2.5^th^ - 97.5^th^ percentiles)
CL/F (L/h)	Clearance of healthy volunteer	6.48 (15.7)	6.34 (4.89 - 7.38)
	Clearance of patient	8.35 (15.8)	8.25 (5.47 - 10.9)
V_2_/F(L)	Central volume	19.7 (15.8)	19.2 (12.3 - 24.3)
K_a_ (1/h)	Absorption rate constant	0.46 (11.6)	0.462 (0.373 - 0.525)
Q /F (L/h)	Inter-compartmental clearance	7.64 (10.8)	7.51 (5.55 - 9.19)
V_3_/F(L)	Peripheral compartment volume	71.8 (18.9)	71.8 (51.7 - 88.6)
ALAG (h)	Absorption lag time	0.168 (14.8)	0.169 (0.123 - 0.196)
F_15mg_	Relative bioavailability of 15 mg	1 FIX	1 FIX
F_10mg_	Relative bioavailability of 10 mg	1.363 FIX	1.36 FIX
F_20mg_	Relative bioavailability of 20 mg	0.537 (15.6)	0.524 (0.413 - 0.609)
*CL_crcl*	The influence of CRCL on CL	1.53 (11.9)	1.53 (1 - 1.97)
*CL_A642*	The influence of genotype of ABCB1 rs1045642 (AA) on CL	0.815 (7.29)	0.821 (0.615 - 0.96)
*IIV_CL*	IIV of CL (%)	35.5 (6.69)	34.8 (25 - 41)
*IIV_V*_*2*_	IIV of V_2_ (%)	59.2 (13.2)	57.7 (19.7 - 72.5)
*IIV_K*_*a*_	IIV of K_a_ (%)	27.8 (40.9)	24.2 (0.313 - 40.2)
*IIV_Q*	IIV of Q (%)	64 (14)	63.1 (48.2 - 73.5)
*IIV_V*_*3*_	IIV of V_3_ (%)	65.9 (11.4)	64.6 (41.6 - 79.6)
*IIV_ALAG*	IIV of ALAG (%)	77.6 (13.7)	76.2 (52.2 - 95.4)
*δ*	Proportional residual error (%)	26.3 (1.32)	26.1 (20.4 - 29.7)

### 3.4 Influence of covariates on rivaroxaban pharmacokinetics

**FIGURE 3:**
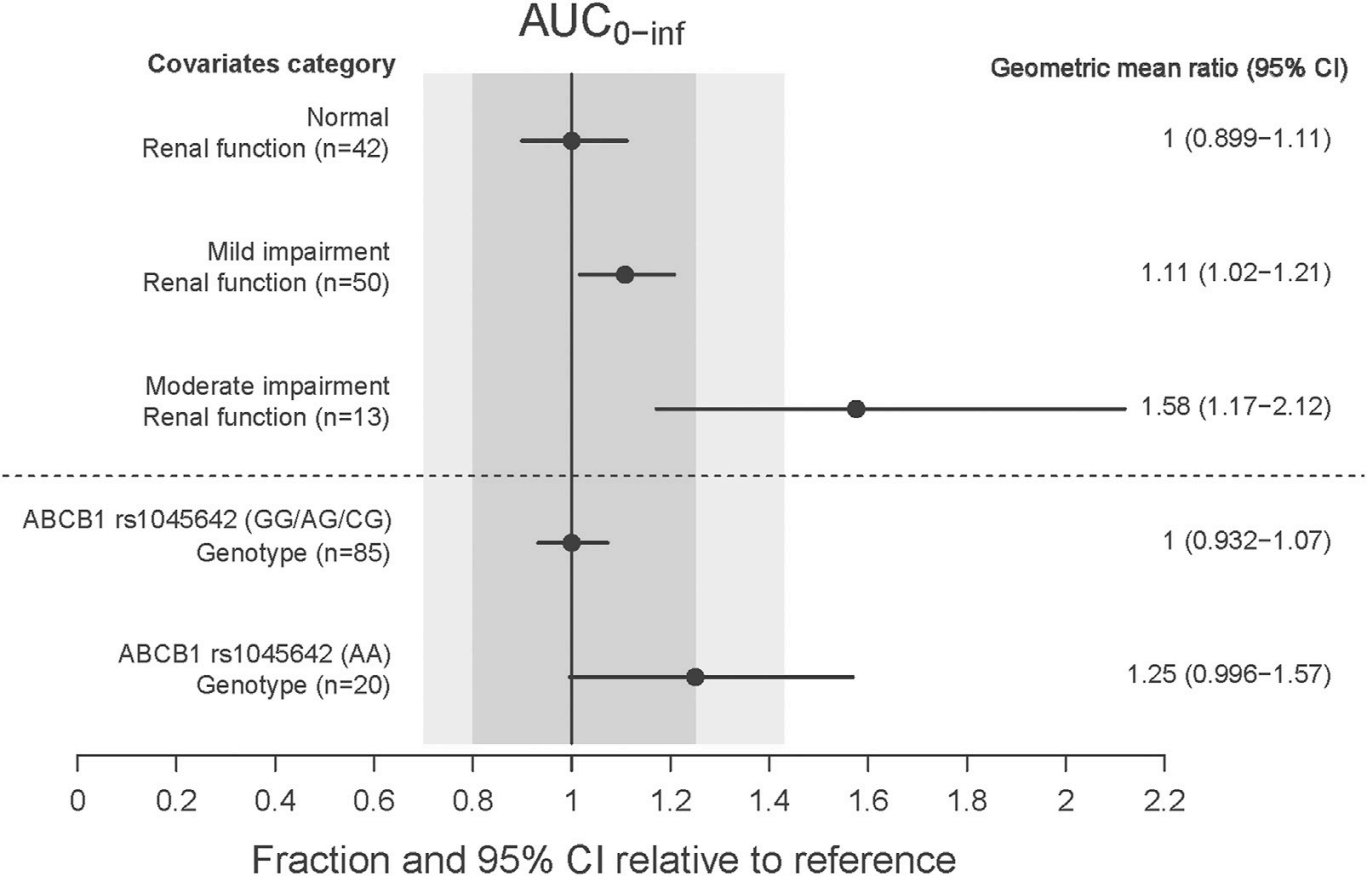
The effect of significant covariates on rivaroxaban exposure (AUC_0–inf_).

**FIGURE 4:**
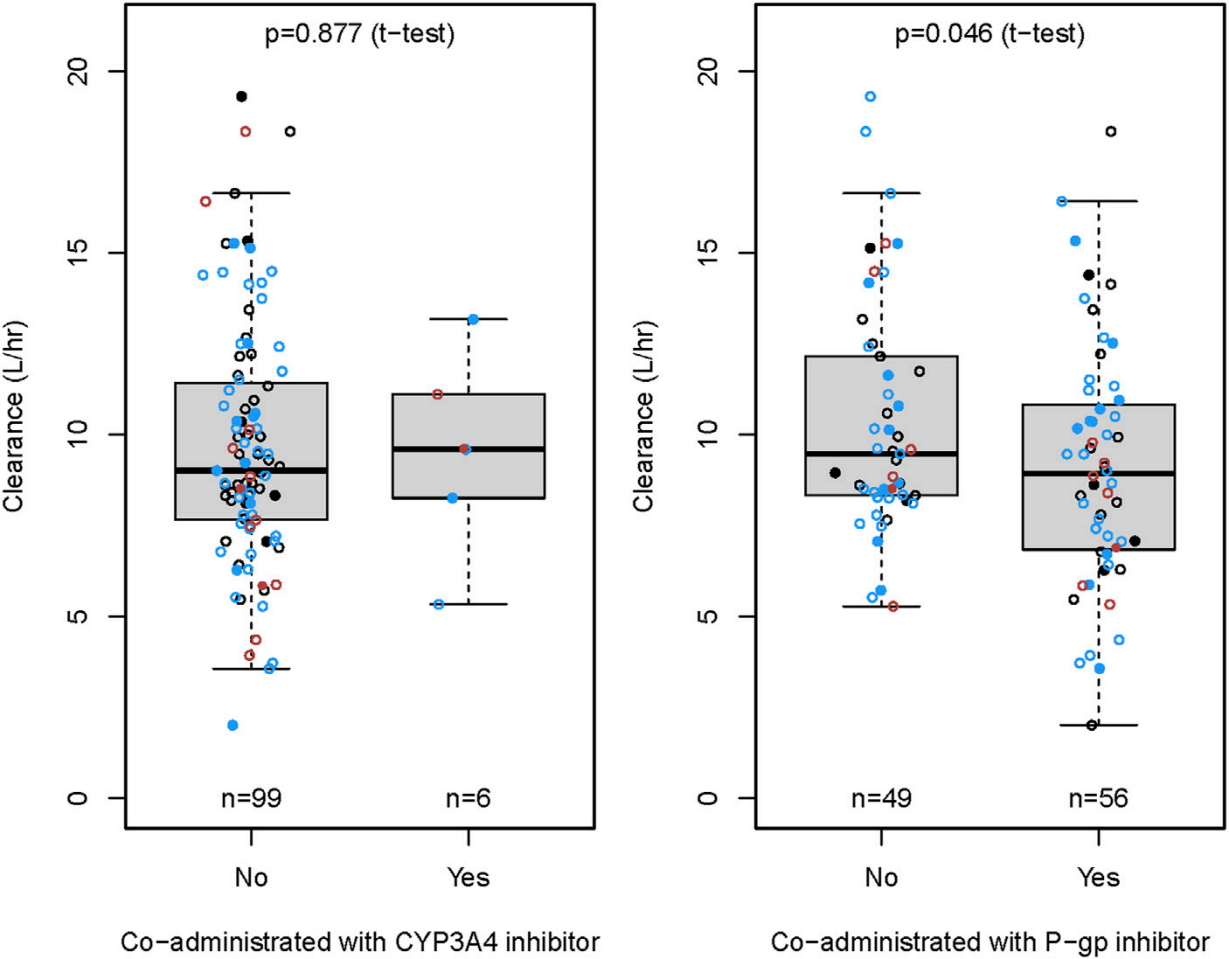
The effect of concomitant drugs on the clearance rate of rivaroxaban.

The CRCL and ABCB1 rs1045642 polymorphism significantly influenced the CL in the final model. The effects on patients with varying degrees of renal impairment in comparison to normal subjects, as well as the impact of the ABCB1 rs1045642 AA genotype relative to other genotypes, are illustrated in the forest plot (Figure 3). This figure indicates that the AUC_0-inf_ increased by 11% and 58% for patients with mild and moderate renal impairment, respectively, compared to normal subjects. Patients with moderately impaired renal function may require a lower dose of rivaroxaban to avoid overexposure. Additionally, the AUC_0-inf_ was observed to increase by 25% for patients possessing the ABCB1 rs1045642 AA genotype when compared to other patient groups. The effects on the clearance of rivaroxaban by patients co-administrated with P-gp inhibitor or CYP3A4 inhibitor are illustrated in the box plot (Figure 4). CL significantly decreased for patients co-administrated with a P-gp inhibitor (*P* < 0.05).

## 4 Discussion

In this study, PK data of rivaroxaban from studies involving healthy volunteers and patients with radiofrequency ablation of NVAF in China were analyzed. Our study first developed a PopPK model of rivaroxaban in both healthy volunteers and patients with radiofrequency ablation of NVAF. They were adequately characterized using a two-compartment model characterized by first-order absorption and first-order linear elimination. CRCL, ABCB1 rs1045642, and the morbid state had a statistically significant effect on the CL of rivaroxaban.

In previous studies, many rivaroxaban PPK models were established with different diseases in several ethnicities, including NVAF, ACS, venous thrombosis, and hip/knee replacement surgery (Zdovc et al., 2019; Mueck et al., 2008; Mueck et al., 2011; Zhang et al., 2022; Liu et al., 2022). For Chinese NVAF patients, Zhang et al. established a PopPK model of rivaroxaban in patients with NVAF in China and quantitatively evaluated the influence of physiological indicators, liver and kidney function, combined disease, combined medication, and genetic factors on the PK of rivaroxaban (Zhang et al., 2022). Another study generated a PopPK model using data from healthy volunteers and investigated using a model based on a mixed healthy volunteer/NVAF patient population. However, quantitatively evaluating the influence of genetics was not included in this research (Zhao et al., 2022). Our study incorporated a complete data set of pharmacokinetic studies in healthy Chinese volunteers and sparse data from patients with radiofrequency ablation of NVAF to optimize a PopPK model of rivaroxaban. Incorporating both healthy volunteer data and patient data into the PopPK model enables robust parameter estimation by leveraging complementary information, thereby enhancing the model’s predictive accuracy across diverse populations. For both healthy volunteers and NVAF patients, the blood samples were collected during hospitalization, and the concentrations of rivaroxaban were determined by LC-MS/MS. Demographic, genetic, and clinical data and concomitant medication on drug PK parameters were quantitatively analyzed, and the significant covariates affecting rivaroxaban PK were identified.

The typical value of CL/F in Chinese NVAF patients estimated in our study was 8.35 L/h, which was similar to the previous results in Chinese NVAF patients (Zhao et al., 2022) but was higher than that in elderly Chinese NVAF patients (3.68 L/h) (Zhang et al., 2023) and in Japanese and Thai NVAF patients (4.72 L/h and 4.19 L/h) ( Singkham et al., 2022). The mean value of V (V_2_ + V_3_) estimated in our study was also higher than in Japanese and Thai patients.

Renal function was reported to be a significant covariate for CL/F in the previous population PK models (Chan et al., 2019). It significantly affected the PK of rivaroxaban due to approximately 35% of rivaroxaban being excreted renally. In a previous study, the AUC of rivaroxaban in German patients with mild and moderate renal impairment were 1.44 and 1.52 times that in patients with normal renal function (Kubitza et al., 2010). For Chinese NVAF patients, Zhang et al. reported that the AUC_0-24, ss_ of rivaroxaban in patients with a CrCl of 50 mL/min or 30 mL/min was 1.2 and 1.3 times than that in patients with a CrCl of 80 mL/min, respectively. In our study, CRCL was one of the significant covariates influencing the CL in the final population model. The results of the covariate impact analysis revealed that patients with mild renal impairment demonstrated an 11% increase in AUC_0-inf_ compared to normal subjects, a change considered minor and not requiring special attention. In contrast, patients with moderate renal impairment showed a 58% increase in exposure relative to subjects with normal renal function. This finding was consistent with those of a previous study in a Caucasian population, which indicated a 1.52-fold (90% confidence interval 1.15–2.01) increase in AUC_24,ss_ for patients with eGFR 30–49 mL/min (Kubitza et al., 2010). Liu et al. reported that 15 mg for Chinese patients with eGFR ≥50 mL/min and normal liver function yielded an exposure comparable to 20 mg for Caucasian patients. We recommended that Chinese patients with moderately impaired renal function may require a lower dose of rivaroxaban to avoid overexposure.

Rivaroxaban exhibited excellent oral bioavailability, with values ranging from 80% to 100% at doses of 10 mg, which remained unaffected by food. Bioavailability for 15 mg and 20 mg doses was 66%, yet rivaroxaban achieved high bioavailability (approximately 80%) when taken with food (Mueck et al., 2014). Our final PopPK model results also indicated that, as the dose increased, the relative bioavailability of the drug decreased. The relative bioavailability of 10 mg rivaroxaban was 1.36-fold that of 15 mg, whereas the relative bioavailability of 15 mg rivaroxaban was 1.86-fold that of 20 mg. In previous research, the relative bioavailability of 15 mg rivaroxaban was 1.43-fold that of 20 mg in Chinese patients with NVAF (Zhao et al., 2022), which was similar to the findings of our study. We speculated that this may be caused by the limited aqueous solubility of a Biopharmaceutical Classification System class II substance (Kushwah et al., 2021). In our study, AUC_0-inf_ was calculated using the formula dose/CL, with all subjects receiving a consistent dose of 15 mg. The AUC_0-inf_ for NAVF patients were 1,198 ng/mL*h, 1,796 ng/mL*h, and 2,395 ng/mL*h for the doses of 10 mg, 15 mg, and 20 mg, which were consistent with a previous study (Zhao et al., 2022). No adverse events were reported after a 3-month follow-up of the NAVF patients. We believed that rivaroxaban dosing could be more effective and/or safer for more patients if increased dosing precision was available through more real-world research.

Several PopPK studies have explored the genetic polymorphisms of rivaroxaban in relation to P-gp, with a particular focus on the ABCB1 gene. Zdovc et al. showed that ABCB1 expression was associated with CL/F, but a relationship between ABCB1 gene polymorphisms and the PK variability of rivaroxaban was not found (Zdovc et al., 2019). Liu et al. indicated that the ABCB1 rs4148738 genotype was statistically significantly associated with CL/F but without clinical relevance (<20%) (Liu et al., 2022). Zhang et al. showed that the ABCB1 rs4728709 mutation was significantly associated with the CL/F and the AUC_24, ss_ (Zhang et al., 2022). Zhang et al. reported that the mutant genotype of ABCB1 rs1045642 significantly increased the CL/F, and elderly patients with a wild genotype of ABCB1 rs1045642 may have had a higher rivaroxaban exposure (Zhang et al., 2023). In our research, the AUC_0-inf_ for patients with the wild genotype of ABCB1 rs1045642 was 25% higher than that for other genotypes, but this effect was deemed moderate and was not expected to have clinical significance. We recommended that Chinese patients with the mutant genotype of ABCB1 rs1045642 should receive a 15 mg dose of rivaroxaban to achieve an effective exposure level.

The NVAF patients frequently presented with comorbid conditions, including hypertension, diabetes, hyperlipidemia, arrhythmias, and coronary heart disease. Consequently, rivaroxaban was commonly administered in conjunction with antihypertensive, hypoglycemic, and antiarrhythmic medications. Our study demonstrated that the CL significantly decreased in patients co-administered with P-gp inhibitors, including amiodarone, propafenone, and felodipine, potentially leading to increased rivaroxaban exposure.

Nevertheless, the present study had several limitations. Most importantly, the amount of patient data used for model construction was limited, and only one real-world, single-center study involved patients, which made it inadequate to quantify the impact of disease-related covariates. Second, another limitation was the population we analyzed was all Chinese individuals. Any ethnic differences in the pharmacokinetics of rivaroxaban between Chinese people and people of other races were not clear.

## 5 Conclusion

In conclusion, the current study established a population pharmacokinetics model of rivaroxaban with a two-compartment model characterized by first-order absorption and first-order linear elimination. The results indicated that creatinine clearance, ABCB1 rs1045642, and morbid state demonstrated the most significant impact on central clearance rate. The PopPK model was expected to help provide relevant PK parameters and covariates information for further studies of rivaroxaban. The study indicated that a daily dose of 15 mg may be appropriate as the primary dosage of rivaroxaban for Chinese patients with NVAF. A lower dose is recommended for patients with moderate renal impairment to avoid overexposure.

## Data Availability

The original contributions presented in the study are included in the article/Supplementary Material; further inquiries can be directed to the corresponding author.
