# Supplementary material for: Population pharmacokinetic analysis of rivaroxaban in healthy volunteers and patients with radiofrequency ablation of non-valvular atrial fibrillation in China
Source: Front Pharmacol. 2025 May 29;16:1562259. doi: 10.3389/fphar.2025.1562259 (PMC12158726; doi:10.3389/fphar.2025.1562259)
Supplement: Supplementary file 1 [file Table1.docx]

TABLE S1 Specific primers used in Sanger sequencing analysis

| Gene | variant | Primer sequence (5' to 3') |
| --- | --- | --- |
| CYP3A4 | rs2242480 | F: ACCAGAGCCAGCACGTTTTA |
|  |  | R: GCCCACATTCTCGAAGACCT |
|  | rs2246709 | F: ACGAAGAAGGGCAAACTAAGC |
|  |  | R: CTCAACAATCCACAAGACCCC |
|  | rs3735451 | F: ATTCAGGCTCCACTTACGGT |
|  |  | R:TCCATGAATTTGTCTCAGGCC |
| CYP3A5 | rs776746 | F: ACTGTTCTGATCACGTCGGG |
|  |  | R: CCACGTATGTACCACCCAGC |
| ABCB1 | rs1045642 | F: GAAGTGTGGCCAGATGCTTG |
|  |  | R: TCAAAGTGTGCTGGTCCTGA |
|  | rs1128503 | F: TGCATCAGCTGGACTGTTGT |
|  |  | R: ACAGCTATTCGAAGAGTGGGC |
|  | rs2032582 | F: GTCCAAGAACTGGCTTTGCTAC |
|  |  | R: TTGCAGGCTATAGGTTCCAGG |
|  | rs4148738 | F: ACCCCTCGGCTTCATTTGAT |
|  |  | R: AAGACACCTCAAACTTGGCC |
|  | rs4728709 | F: TCATATCCCAGCTCTGAGTCC |
|  |  | R:ACATGAGTTCAAGCACAGTCA |
| ABCG2 | rs3114018 | F: ATTTGGGTGTCTGAGGGGAG |
|  |  | R: CTAAGGCCCTGTATCTCCCG |
